# Low birth weight in a sub-urban area of Cameroon: an analysis of the clinical cut-off, incidence, predictors and complications

**DOI:** 10.1186/s12884-015-0723-9

**Published:** 2015-11-04

**Authors:** Tsi Njim, Julius Atashili, Robinson Mbu, Simeon-Pierre Choukem

**Affiliations:** Department of Internal Medicine and Pediatrics, Faculty of Health Sciences, University of Buea, Buea, Cameroon; Department of Public Health and Hygiene, Faculty of Health Sciences, University of Buea, Buea, Cameroon; Department of Obstetrics and Gynaecology, Faculty of Medicine and Biomedical Sciences, University of Yaounde, Yaounde, Cameroon; Health and Human Development (2HD) Research Group, Douala, Cameroon; Diabetes and Endocrine Unit, Department of Internal Medicine, Douala General Hospital, Douala, Cameroon

**Keywords:** Low birth weight, Incidence, Cut-off value, Predictor, Complications, Cameroon

## Abstract

**Background:**

The World Health Organisation recommends that each country adopts its own cut-off value of low birth weight (LBW) for clinical use. The aims of this study were to establish a clinical cut-off point for LBW and to determine its incidence, predictors and complications in a sub-urban area’s hospital of Cameroon.

**Methods:**

We conducted a study in two phases: a 6-year retrospective phase during which we collected demographic and clinical information from the records of the maternity of the Buea Regional Hospital (BRH) and a 3-month prospective phase during which data were collected from consenting pregnant women using a structured questionnaire, and newborns were examined and followed after birth.

**Results:**

A total of 4941 records were reviewed during the retrospective phase and the 10^th^ centile of birth weights was 2600 g. In the 200 pregnant women enrolled during the prospective phase, using this cut-off yielded an incidence of LBW of 19.0 %. Independent predictors of LBW were preterm delivery, hypertensive disorders in pregnancy, HIV infection, maternal age >36 years, maternal height <150 cm and pre-delivery BMI < 25 kg/m^2^. Neonates with LBW were more likely to have neonatal asphyxia, foetal distress, respiratory distress and neonatal death.

**Conclusions:**

Our results suggest that newborns under 2600 g have LBW in sub-urban Cameroon. They represent one out of every five babies, and they deserve close care. Preventive measures targeting the predictors described here are warranted to reduce the incidence and complications. Similar studies in urban areas are required in order to generalize the results.

## Background

Low birth weight (LBW) is weight of a newborn lower than the 10^th^ centile for its gestation [1]. The World Health Organisation (WHO) estimates that 15.5 % (over 20 million) of all newborns worldwide have LBW, more than 95 % of whom reside in the developing world [2]. LBW is associated with up to 60-80 % of neonatal deaths in these developing countries [3].

The 10^th^ centile used to define LBW is usually set at 2500 g [2], a cut-off value generated from epidemiological observations that morbidity and mortality increases in newborns who have birth weights below 2500 g. However, the WHO recommends using this value mainly for comparative health statistics, not for clinical care. It suggests that for clinical purposes, individual countries should adopt alternative figures [2]. Despite the recommendation, most developing countries including Cameroon still use 2500 g to define LBW in clinical practice. This has public health implications because since LBW is a serious health problem in these countries, some newborns with LBW may not benefit from adequate care if an inappropriately lower value were used as a cut-off point, or an unbearable financial burden could result from the use of a cut-off point higher than what it should actually be.

South Central Asia bears the highest burden of LBW infants – with about 27 % of all birth. The UNICEF/WHO estimates report an incidence of LBW of 11 % in Cameroon [2]. A recent study in 2012 in the Buea Health District (BHD) reported a prevalence of 13.7 % [4]. This high prevalence of LBW constitutes a public health problem as LBW is usually an indicator of chronic maternal malnutrition, maternal illness and poor prenatal care, hence, a good indicator of the socioeconomic status [2].

In Cameroon, studies to define a cut-off value that can be used for clinical care have not yet been carried out to the best of our knowledge. In this study we sought to determine the clinical cut-off value for LBW in a sub-urban area’s hospital. We also assessed the incidence, predictors and complications of LBW.

## Methods

### Study design and setting

We carried out a descriptive and analytic study with two phases at the Buea Regional Hospital (BRH): a retrospective register analysis used to determine the cut-off of LBW in our population, and a prospective phase used to assess the incidence, predictors and complications of LBW.

The BRH is situated in the Buea Health District (BHD) which is part of the Fako Division, South-West Region, Cameroon. The BRH acts as the reference hospital in the health district and also performs more deliveries than all the other health facilities in the district. The hospital has all standard units of general medicine. One gynaecologist-obstetrician and one paediatrician run the maternity and the paediatric units respectively. The institution lacks a neonatal intensive care unit; also, the maternity is situated 114 m apart from the operating rooms (where oxygen is kept). This distance represents a pitfall whenever a newborn requires oxygen.

### Participants and sampling

**Fig. 1:**
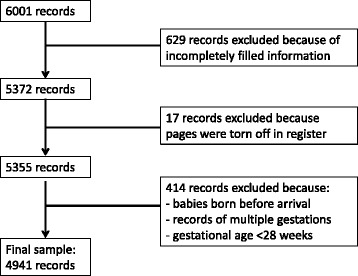
Reasons for exclusion of records in the retrospective phase. From the 1^st^ of January, 2007 to the 31^st^ of December, 2012, 6001 deliveries occurred in the BRH. From this amount of deliveries, 4941 records were included in the retrospective phase of the study yielding a response rate of 82.3 %. The various reasons for the exclusion of some records included: incomplete records (629), destroyed records (17) and babies born before arrival at the hospital, abortions and multiple gestations (414)

For the retrospective phase, all the records of pregnant women who gave birth during a six year period from the 1^st^ January 2007 to the 31^st^ December 2012 were reviewed. Only the records that clearly stated a gestational age less than 28 completed weeks, reported multiple gestation or had incomplete information were excluded (Fig. 1). Information was said to be complete if it contained: maternal age, marital status, gestational age, type of delivery, birth weight, sex of the newborn and APGAR score at the first minute. These records were collected and carefully stored in a handwritten register by the obstetrician and midwives in the maternity.

**Fig. 2:**
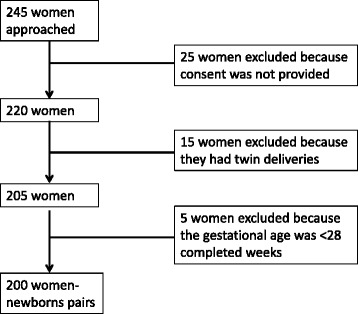
Reasons for the exclusion of some pregnant women in the prospective phase. From the 2^nd^ of January, 2013 to the 23^rd^ of March, 2013, the period during which the prospective phase of the study took place, a total of 245 deliveries took place in the BRH. The study included 200 of these women and their babies yielding a response rate of 88.9 % from the prospective phase of the study. The various reasons for the exclusion of some pregnant women-neonate pairs were: multiple gestations (15), abortions (5) and refusal to provide consent (25)

For the prospective phase, the target study population included all pregnant women-newborn pairs who attended the BRH during the period of the study; we excluded women who delivered at a gestational age below 28 weeks, those who had multiple gestations and those who did not provide consent to take part in the study (Fig. 2).

### Data collection, variables and measurements

In the retrospective phase, after obtaining ethical clearance from the Institutional Review Board of the University of Buea, we obtained permission to access the birth records in the BRH from the Regional Delegate of Public Health for the South west region and the Director of the BRH. Socio-demographic and clinical data were collected from the birth records of the hospital. The birth weights of infants born in the hospital during the defined period were recorded. These data had been collected by midwives, trained nurses and doctors who worked in the maternity of the hospital. These data were handwritten into a register stored in the maternity. A total of 4941 records was included in this phase.

The prospective phase was carried out over a period of 3 months, from January 2 to March 23, 2013. Pregnant women who were about to put to birth had their labour monitored making sure that they were provided the standard care that is expected.

Participants’ hospital records were used to extract information on the following

- Socio-demographic characteristics including socioeconomic status, and level of education further divided into high (secondary and tertiary) and low (none and primary).
- Obstetric history (gravidity, parity, year of first pregnancy, previous birth weight, antenatal care (ANC) history for the current pregnancy, and history of anaemia, fever, malaria and urinary tract infections during the current pregnancy.
- Past medical history (history of chronic illness like HIV, hypertension and chronic liver disease, history of pregnancy related illnesses like pre-eclampsia/eclampsia and gestational hypertension, and pre-pregnancy weight).
- Toxicological history (alcohol intake, cigarette smoking, other substance abuse and recreational drug use).

Participants’ weight -to the nearest kg using a *Camry bathroom scale*-, height -to the nearest cm using a locally made stadiometer calibrated to the *Butterfly brand* measuring tape- were measured, and a standard physical examination was performed. Throughout the labour, the foetus was monitored for the presence of acute foetal distress (foetal heart rate < 120 beats per minute and/or green meconium-stained amniotic fluid at delivery).

After delivery, the neonates were assessed for viability (breathing, beating of the heart, pulsation of the umbilical cord or definite movements of voluntary muscles), and their APGAR scores at the first, fifth and tenth minutes were determined. The baby’s weight was measured to the nearest gram using a *Holtex + digital* baby scale. The newborns were monitored for the presence of respiratory distress which was identified by the presence of any one of the following: respiratory grunting, nasal flaring, intercostal recessions, sub-xyphoid recession, thoraco-abdominal asynchrony, and respiratory rate < 40 or > 60 breaths per minute. All stillbirths and any in-hospital neonatal death was noted.

The mother was then approached and informed of the entire study and her consent requested. Only those who agreed to participate in the study were included after they signed the inform consent form. Minors and their guardians signed assent and guardian consent forms respectively. The newborns and their mothers were observed until the day of discharge. This was usually a period of 3 days for normal deliveries and could extend to 10 days for complicated deliveries including caesarean sections and instrumental deliveries. Two hundred participants were enrolled during this prospective phase.

### Data management and statistical analysis

Data were cross-checked for errors before entry into a password-protected personal computer. The data were stored securely in a private location and kept confidential. Participants were referred to only by identification numbers. Identifiable information (consent forms) was kept separate from the data collection forms and it was only possible to link both through a coding sheet which was available solely to the principal investigator.

Data were analysed using *Epi Info version 3.5.4* and *Microsoft Excel 2007.* Means (standard deviations) were used to summarise continuous variables, and proportions and frequencies for categorical variables. Frequencies were compared using Fisher’s exact tests. The McNemar test was used to compare the incidence of LBW using the traditional cut-off (2500 g) to that yielded by our newly identified cut-off, and the agreement between the two was also computed. Predictors for LBW were determined using bivariate and multiple logistic regression models. The multivariate model included all variables which p-values in the bivariate analyses were less than or equal to 0.25 [5]. The statistical significance was set at p < 0.05.

### Ethical considerations

Ethical approval was obtained from the Institutional Review Board of the Faculty of Health Sciences, University of Buea. The study also received administrative clearance from the South-West Regional Delegation of Public Health and from the Director of the BRH.

## Results

### Socio-demographic and obstetrical characteristics of mothers

The mean age of the mothers was 26.4 ± 5.5 and 26.4 ± 5.8 years respectively for the retrospective and prospective phases of the study. Most were married, half were unemployed, almost all of them had received at least a primary level of education and 6.6 % of the women reported spending less than a USD (United States dollar) a day (Table 1). None of the women admitted to have ever smoked cigarettes or used any recreational drug.

**Table 1:** Socio-demographic characteristics and obstetric history of mothers delivering in the Buea Regional Hospital, January – March 2013

Socio-demographic factor	Groups	Number	Frequency (%)
Marital status (*N* = 200)	Married	114	57.0
	Single	85	42. 5
	Widow	1	0. 5
Occupation (*N* = 200)	Unemployed	101	50. 5
	Employed	38	19.0
	Self-employed	61	30.5
Level of education (*N* = 199)	None	2	1.0
	Primary	38	19.1
	Secondary first cycle	70	35.2
	Secondary second cycle	28	14.1
	Tertiary	61	30.7
Age groups (*N* = 200)	15-19	30	15.0
	20-36	159	79.5
	37-44	11	5.5
Gravidity (*N* = 200)	<5	171	85.5
	≥5	29	14.5
Parity (*N* = 200)	Nulliparous (parity = 0)	90	45.0
	Multiparous (parity > 1)	110	55.0

Regarding the obstetrical characteristics in the prospective phase (Table 1), 34.5 % were primigravidas, 45.0 % were nulliparous, 98.0 % of the pregnant women had never experienced a preterm delivery and 46.0 % did not have any living child.

### Distribution of birth weight and incidence of low birth weight

**Fig. 3:**
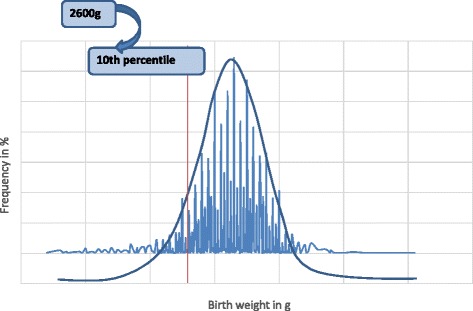
Distribution of birth weights (*n* = 4941). The bell-shaped blue curve shows that the birth weights in the BRH have a Gaussian distribution. The red line corresponds to the 10^th^ percentile of birth weights in our population (2600 g) while the blue lines show the frequencies for the various birth weights

The 10^th^ centile of birth weight was 2600 g (Fig. 3), which corresponds to the cut-off to define LBW in our newborn population. In the prospective phase 38 babies had birth weights below 2600 g, giving an incidence of 19.0 % (95 % CI: 13.8, 25.1). Using the traditional cut-off of 2500 g, the incidence was 13.5 % (95%CI: 9.1, 19.0). The difference between the two incidences was statistically significant (p = 0.001). However, the agreement rate between the two was high, 94.5 % (Kappa = 0.80).

### Predictors of low birth weight

In bivariate analysis, factors significantly associated with LBW were the following: socio-demographic factors: maternal age >36 years (*p* = 0.04) (Table 2); obstetrical and clinical factors: preterm deliveries (p < 0.001), number of ANC visits < 4 (*p* = 0.03), maternal HIV infection (*p* = 0.02) and hypertensive disorders in pregnancy (p < 0.01) (Table 3); anthropometric factors: weight gain during pregnancy ≤10 kg (p < 0.01) and pre-delivery Body mass index (BMI) <25 kg/m^2^ (p < 0.01) (Table 4).

**Table 2:** Bivariate analysis of potential socio-demographic predictors of low birth weight in deliveries in the Buea Regional Hospital, January – March 2013

Predictors		Total	LBW	LBW	Odds ratio	95 % CI	*p* value
		*N*	*n*	(%)			
Marital status (*N* = 200)	Married	114	21	18.4	1.0		
	Single	86	17	19.8	1.1	0.5 - 2.2	0.47
Occupation (*N* = 200)	Employed	99	22	22.2	1.0		
	Unemployed	101	16	15.8	0.7	0.3 – 1.5	0.17
Teenage mother (*N* = 200)	Non-teens	170	32	18.8	1.0		
	Teenagers	30	6	20.0	1.1	0.4 – 2.9	0.52
Maternal age (*N* = 200)	≤ 36 years	189	33	17.5	1.0		
	> 36 years	11	5	45.5	3.9	1.1 – 13.7	0.04
Level of education (*N* = 199)	High	61	8	13.1	1.0		
	Low	138	30	21.7	1.8	0.8 – 4.3	0.11
Average amount spent per day (*N* = 182)	≥ 1USD	170	32	18.8	1.0		
	< 1USD	12	2	16.7	0.8	0.2 – 4.1	0.60
Alcohol consumption (*N* = 200)	No	148	27	18.2	1.0		
	Yes	52	11	21.2	1.2	0.5 – 2.6	0.39
Alcohol consumption during pregnancy (*N* = 200)	No	160	30	18.8	1.0		
	Yes	40	8	20.0	1.1	0.5 – 2.6	0.51

*LBW* Low Birth Weight, *USD* United States Dollars

**Table 3:** Bivariate analysis of potential obstetric and clinical predictors of low birth weight in deliveries in the Buea Regional Hospital, January – March 2013

Predictors		Total	LBW	LBW	Odds ratio	95 % CI	*p*-value
		*N*	*n*	(%)			
Gravidity (*N* = 200)	< 5	171	29	17.0	1.0		
	≥ 5	29	9	31.0	2.2	0.9 – 5.3	0.07
Gestational age (*N* = 200)	Term	163	19	11.7	1.0		
	Preterm	37	19	51.4	8.0	3.6 – 17.9	<0.01
Previous LBW (*N* = 200)	No	193	35	18.1	1.0		
	Yes	7	3	42.9	3.4	0.7 – 15.8	0.12
Number of ANC visits (*N* = 189)	≥ 4	115	15	13.0	1.0		
	< 4	76	19	25.0	2.2	1.0 – 4.7	0.03
Malaria in pregnancy (*N* = 200)	No	144	24	16.7	1.0		
	Yes	56	14	25.0	1.7	0.8 – 3.5	0.13
Anaemia in pregnancy (*N* = 163)	No	90	15	16.7	1.0		
	Yes	73	16	21.9	1.4	0.6 – 3.1	0.26
Hypertensive disorders in pregnancy (*N* = 200)	No	187	30	16.0	1.0		
	Yes	13	8	61.5	8.4	2.6 – 27.3	<0.01
HIV infection (*N* = 200)	No	183	31	16.9	1.0		
	Yes	17	7	41.2	3.4	1.2 – 9.7	0.02

*ANC* Antenatal Care, *LBW* Low Birth Weight

**Table 4:** Bivariate analysis of potential anthropometric predictors of low birth weight in deliveries in the Buea Regional Hospital, January – March 2013

Predictors		Total	LBW	LBW	Odds ratio	95 % CI	*p*-value
		N	n	(%)			
Maternal height (*N* = 199)	≥ 150 cm	190	33	17.4	1.0		
	< 150 cm	9	4	44.4	3.8	1.0 – 14.9	0.06
Weight gain in pregnancy (*N* = 131)	> 10 kg	85	10	11.8	1.0		
	≤ 10 kg	46	16	34.8	4.0	1.6 – 9.8	<0.01
Pre-delivery BMI (*N* = 199)	≥ 25 kg/m^2^	169	24	14.2	1.0		
	< 25 kg/m^2^	30	13	43.3	4.6	2.0 – 10.7	<0.01

*LBW* Low Birth Weight, *BMI* Body mass index

Malaria or anaemia in pregnancy, gravidity ≥ 5, being a teenager, single marital status, alcohol consumption before or during pregnancy, a history of a previous LBW, being unemployed, maternal height < 150 cm, spending < 1USD a day and a low level of education were not associated with LBW (Tables 2, 3 and 4).

In multivariate analysis the following factors were independently associated with LBW: preterm delivery (aOR = 9.3, 95 % CI 1.9 - 44.8; p < 0.01), maternal height < 150 cm (aOR = 13.6, 95 % CI 1.1 – 175.4; p = 0.05), maternal HIV infection (aOR = 11.2, 95 % CI 1.5 – 82.6; *p* = 0.02), hypertensive disorders in pregnancy (aOR = 17.4, 95 % CI 2.3 - 133.3; p < 0.01), maternal age > 36 years (aOR = 14.5, 95 % CI 1.7 - 123.5; *p* = 0.01) and pre-delivery BMI < 25 kg/m^2^ (aOR = 10.4, 95 % CI 1.5 - 70.4; *p* = 0.02).

### Complications of LBW

Neonates with LBW had a significantly higher risk of developing the following complications (Table 5): neonatal asphyxia by the 5^th^ minute (*p* = 0.02) and 10^th^ minute (*p* = 0.03), foetal distress during labour (*p* = 0.01) and respiratory distress after birth (p < 0.01). They were also four times more likely to die within the early neonatal period (p < 0.01). We also observed that neonates who fell within the category >2500 g and ≤2600 g had significantly higher prevalence of complications compared with those > 2600 g (Table 6).

**Table 5:** Complications of low birth weight in deliveries in the Buea Regional Hospital, January – March 2013

Complications		LBW		NBW		Total	*p*-value
		*N*	(%)	*N*	(%)		
Neonatal asphyxia (1^st^ minute)	Yes	17	47.2	55	34.6	72	0.10
	No	19	52.8	104	65.4	123	
Neonatal asphyxia (5^th^ minute)	Yes	11	29.7	22	13.7	33	0.02
	No	26	70.3	139	86.3	165	
Neonatal asphyxia (10^th^ minute)	Yes	7	18.9	11	6.8	18	0.03
	No	30	81.1	150	93.2	182	
Foetal distress	Yes	6	21.4	33	19.2	39	0.01
	No	22	78.6	139	80.8	161	
Respiratory distress	Yes	7	18.4	7	4.3	14	< 0.01
	No	31	81.6	155	95.7	186	
Neonatal death	Yes	9	23.7	7	4.3	16	< 0.01
	No	29	76.3	155	95.7	184	

*LBW* Low Birth Weight, *NBW* Normal Birth Weight

**Table 6:** Complications of low birth weight babies within the category >2500 g to ≤2600 g in deliveries in the Buea Regional Hospital, January – March 2013

Complications		LBW2		NBW2		Total	*p*-value
		*N*	(%)	*N*	(%)		
Neonatal asphyxia (5^th^ minute)	Yes	4	40.0	22	13.7	26	0.05
	No	6	60.0	139	86.3	146	
Neonatal asphyxia (10^th^ minute)	Yes	3	30.0	11	6.8	14	0.04
	No	7	70.0	150	93.2	157	
Foetal distress	Yes	4	40.0	26	16.1	30	0.07
	No	6	60.0	136	84.0	142	
Respiratory distress	Yes	1	10.0	7	4.3	8	0.4
	No	9	90.0	155	95.7	164	
Neonatal death	Yes	3	30.0	7	4.3	10	0.01
	No	7	70.0	155	95.7	162	

*LBW2* Low Birth Weight between 2500 g to 2600 g, *NBW2* Normal Birth Weight >2600 g

## Discussion

We found in our study that the 10^th^ centile of birth weight that defines the cut-off point for low birth weight (LBW) was 2600 g. In this sub-urban newborn population the clinical cut-off for LBW was therefore higher than the 2500 g traditionally used elsewhere. We are not aware of any study that has attempted to set the cut-off of LBW in Cameroon or elsewhere in sub-Saharan Africa. In developed countries, cut-off used in studies span from 2750 g in USA in the early 1990s [6] to 3000 g recently in Denmark [7].

Using 2500 g as the cut-off, the incidence of LBW was 13.5 %. A recent study in the same area yielded a similar result (13.7 %) [4]. This incidence is also in agreement with the year 2000 UNICEF/WHO estimates of 13.9 % in Africa, and falls in between the lowest worldwide regional prevalence found in Europe (8.0 %) and the highest that is in South-East Asia (26.2 %) [2]. When 2600 g was used as the reference value, we obtained a significantly higher prevalence of 19 %. In other words, using 2500 g underestimates the burden of LBW and excludes a significant proportion (5.5 %) of newborns with LBW from receiving appropriate care in the study area. This is further strengthened by our observation that newborns between 2500 g and 2600 g of birth weight had higher rates of complications than those with birth weight > 2600 g.

In multivariate analysis we found that preterm delivery, maternal height < 150 cm, maternal HIV infection, hypertensive disorders in pregnancy, maternal age > 36 years, and pre-delivery BMI < 25 kg/m^2^ were independent predictors of LBW. The role of preterm delivery as a predictor of LBW is obvious because preterm babies have not thrived enough to reach their target term weight. These findings reinforce the relevance of appropriate prevention and care of pregnancy-associated comorbidities like HIV infection and hypertensive disorders to prevent LBW. They also emphasize the risk associated with advanced maternal age. Siza et al. in Tanzania also identified hypertensive disorders in pregnancy and HIV infection as factors associated with LBW [8]. The vicious circle of HIV, poverty and maternal illness [9] explains the high incidence of LBW among HIV infected women. It was expected that LBW would be associated with low income as indicated with low estimate of daily expenditure. This was not the case and this could represent difficulty in quantifying daily expenditures in our setting.

Women usually gain weight during pregnancy, and a pre-delivery BMI < 25 kg/m^2^ – a combination of maternal, fetal and amniotic fluid weights - highly suggest a pre-pregnancy undernutrition. Indeed, Lawoyin *et al*. previously reported that mothers who had LBW neonates had significantly lower BMI at the onset of pregnancy and gained significantly less weight in the third trimester and last four weeks of a term pregnancy [10]. This probably explains our finding that women with pre-delivery BMI <25 kg/m^2^ were more likely to have LBW neonates. In Tanzania pregnant women who had malnutrition (BMI <18 kg/m^2^) had the highest proportions of LBW neonates, followed by pregnant women who were undernourished (BMI from 18-22 kg/m^2^) [8]. All the above confirm that a mother with poor nutritional status before and during pregnancy, does not provide adequate nutrients necessary for foetal growth.

The association between maternal socio-economic status indicators such as low level of education or low incomes is controversial in developing countries. Some studies have reported an association with LBW [8], whereas others have not found any association [11]. We also did not observed any association between surrogates of socio-economic status and LBW.

LBW neonates in our study were more prone to foetal distress, low APGAR scores at delivery and respiratory distress than those with normal birth weight. Simiyu *et al*. in Kenya had as leading complications of LBW: respiratory distress, apnoeic spells and sepsis [12]. We also found that LBW babies were considerably more likely to die in the neonatal period. This was similar to what Lawoyin obtained in Nigeria in 2001 [13]. Kouam and Kamdom-Moyo in Yaounde showed that perinatal mortality decreased as the weight of the baby increased [14] while Kedy Koum *et al.* in Douala reported LBW as the most common diagnosis associated with neonatal death [15].

We acknowledge the following potential limitations: our study was carried out in only one health facility, and we did not include a substantial number of women who put to birth at home; thus results might not reflect the situation in the whole Health District nor in all sub-urban areas in Cameroon. Also, the retrospective phase was subjected to a potential risk that some of the records were not correctly filled. Furthermore, some of the risk factors like the mother’s income, history of fever in pregnancy and the level of alcohol consumption were subjective as they were based on the mother’s recall. Some other risk factors like recreational drug use, cigarette smoking during pregnancy may have been overlooked as some women may deliberately deny in order to appear socially virtuous. Nevertheless, this is the first report to the best of our knowledge that sets a cut-off for LBW for local use, on a sample of almost 5000 newborns. Despite the small sample size in the prospective phase, we used robust statistics to identify strong predictors of LBW, that are worth preventing.

## Conclusions

Overall, our results suggest that the definition of LBW in sub-urban areas in Cameroon may need to be raised so as to allow all babies < 2600 g to benefit from close care. However, this finding needs to be confirmed by other studies. One out of every five babies are born with a LBW. These neonates are more likely to suffer from neonatal asphyxia, foetal distress, respiratory distress and death. If the predictors identified in this study were systematically screened during antenatal care and delivery and managed appropriately, this would reduce the morbidity and mortality due to LBW and contribute to the achievement of the Millennium Development Goal 4. Similar studies in urban areas are required in order to generalize the results to the whole country and sub-Saharan Africa, especially because confirming our data wouldl increase the proportion of newborn who deserve special care, with consequences on health expenditures.

## Abbreviations

ANC: Antenatal care
BHD: Buea Health District
BMI: Body mass index
BRH: Buea Regional Hospital
CI: Confidence interval
HIV: Human Immunodeficiency virus
LBW: Low birth weight
UNICEF: United Nation International Children Emergency Fund
USA: United States of America
USD: United states dollar
WHO: World Health Organization
